# P-329. Estimating changes in MRSA infection rates due to changes in facility MRSA precautions

**DOI:** 10.1093/ofid/ofae631.532

**Published:** 2025-01-29

**Authors:** Karim Khader, Candace Haroldsen, Martin Evans, Loretta Simbartl, Brian McCauley, Matthew H Samore, Michael Rubin

**Affiliations:** University of Utah, Salt Lake City, Utah; University of Utah, Salt Lake City, Utah; Veterans Affairs, Lexington, KY; National Infectious Diseases Service, Veterans Health Administration, Cincinnatti, Ohio; National Infectious Diseases Service, Veterans Health Administration, Cincinnatti, Ohio; University of Utah, Salt Lake City, Utah; University of Utah, Salt Lake City, Utah

## Abstract

**Background:**

The role of active surveillance (AS) and contact precautions (CP) in acute care settings for preventing transmission of endemic drug resistant organisms such as methicillin-resistant *Staphylococcus aureus* (MRSA) remains unsettled. The emergence of SARS-CoV-2 led to a shift in VA policy in March 2020, when VA acute care facilities were allowed to suspend AS for MRSA and CP for patients with MRSA colonization or active infection, resulting in a partial and variable de-implementation of AS/CP across the VA (**Figure 1**). We evaluated the impact of this change on VA MRSA infection rates.

**Figure:**
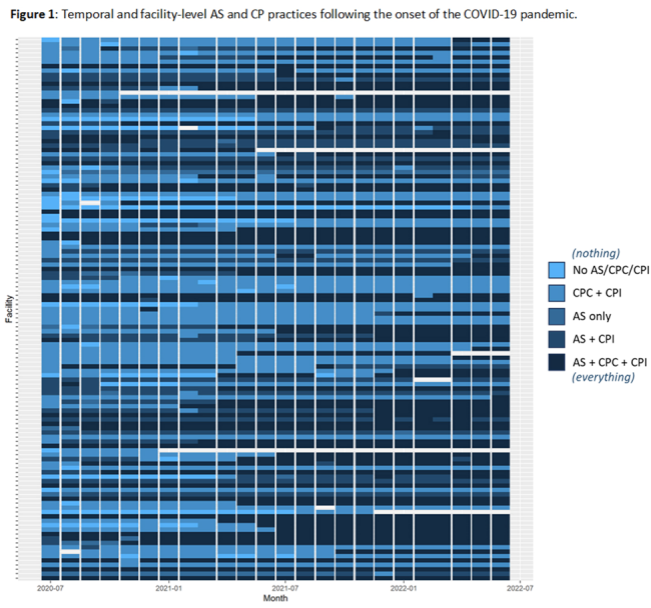

**Methods:**

We modeled monthly positive MRSA culture rate at the ward-facility level using (1) all positive MRSA cultures, and (2) only positive sterile-site cultures (to more closely capture ‘true infections’). Our primary outcome was HAIs (occurring ≥ 3d after admission), including those up to 30-days post-discharge. We used Poisson, Negative Binomial, and Poisson mixed-effects regression approaches, clustering within ward type (ICU/non-ICU) and facility. With each approach, 4 different models incorporated increasing numbers of covariates to evaluate the robustness of the results.

**Figure:**
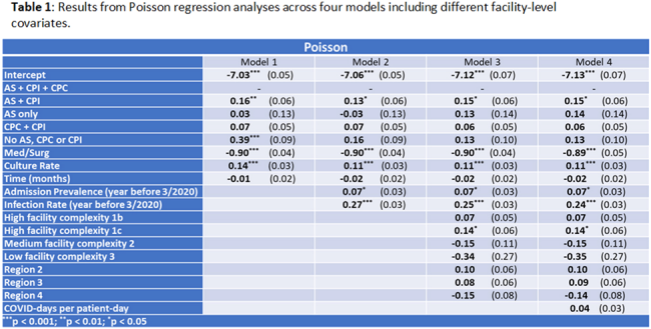

**Results:**

Using Poisson regression, we found culture rate, discontinuing AS & CP, and discontinuing CP only for colonization are associated with an increased MRSA positive culture rate (Model 1, **Table 1**). Addition of the year prior infection rate (Models 2–4) nullifies the association between AS & CP and MRSA positive culture rate. Results were similar when modeling positive sterile site cultures only (**not shown**) and using Negative Binomial regression (**Table 2**). Facility-level use of AS & CP is not associated with MRSA positive culture rate when using Poisson mixed-effects regression (**Table 3**), suggesting that accounting for facility-level clustering eliminates (or reduces) the apparent effect of AS/CP.

**Figure:**
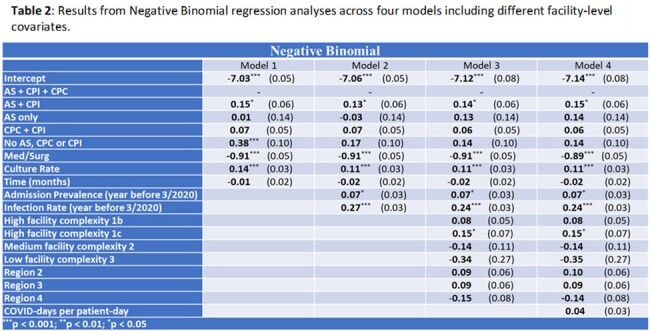

**Conclusion:**

The impact of de-implementing AS & CP is complex and difficult to discern. The inference that de-implementation of AS & CP increased MRSA infection rates was not robust to relaxation of simplifying assumptions. These findings combined with the existence of unobservable data (e.g., use of gowns/gloves during the pandemic), imply substantial challenges surrounding statistical interpretation.

**Figure:**
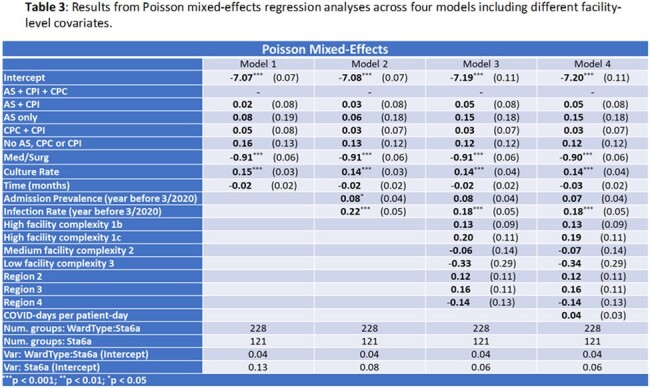

**Disclosures:**

**All Authors**: No reported disclosures

